# Risk Factors for Tuberculosis

**DOI:** 10.1155/2013/828939

**Published:** 2013-02-12

**Authors:** Padmanesan Narasimhan, James Wood, Chandini Raina MacIntyre, Dilip Mathai

**Affiliations:** ^1^School of Public Health and Community Medicine, The University of New South Wales, Kensington, Sydney, NSW 2052, Australia; ^2^Infectious Diseases Research and Training Centre, Department of Medicine-I and Infectious Diseases, Christian Medical College, Vellore, Tamil Nadu, India

## Abstract

The risk of progression from exposure to the tuberculosis bacilli to the development of active disease is a two-stage process governed by both exogenous and endogenous risk factors. Exogenous factors play a key role in accentuating the progression from exposure to infection among which the bacillary load in the sputum and the proximity of an individual to an infectious TB case are key factors. Similarly endogenous factors lead in progression from infection to active TB disease. Along with well-established risk factors (such as human immunodeficiency virus (HIV), malnutrition, and young age), emerging variables such as diabetes, indoor air pollution, alcohol, use of immunosuppressive drugs, and tobacco smoke play a significant role at both the individual and population level. Socioeconomic and behavioral factors are also shown to increase the susceptibility to infection. Specific groups such as health care workers and indigenous population are also at an increased risk of TB infection and disease. This paper summarizes these factors along with health system issues such as the effects of delay in diagnosis of TB in the transmission of the bacilli.

## 1. Introduction

In addition to providing effective treatment and reducing mortality, a primary aim of tuberculosis (TB) control programs in countries of high TB incidence is to reduce the transmission from infectious TB cases. The development of TB in an exposed individual is a two-stage process following infection. In most infected persons, infection is contained by the immune system and bacteria become walled off in caseous granulomas or tubercles. In about 5% of infected cases, rapid progression to tuberculosis will occur within the first two years after infection [1]. About 10% of people with latent infection will reactivate, half within the first year, the remainder over their lifetime [2–7] mostly by reactivation of the dormant tubercle bacilli acquired from primary infection or less frequently by reinfection. Overall, about 10–15% of those infected go on to develop active disease at some stage later in life [2], but the risk of progression is much higher at about 10% per year [8, 9] in HIV-positive and other immunocompromized individuals.

The risk of progression to infection and disease is two different aspects and proper understanding of these factors is essential for planning TB control strategies [10]. The risk of infection following TB exposure is primarily governed by exogenous factors and is determined by an intrinsic combination of the infectiousness of the source case, proximity to contact and social and behavioural risk factors including smoking, alcohol, and indoor air pollution. In settings with increased chances of social mixing (together with overcrowding) transmission will be high. Similarly, conditions which prolong the length of exposure to an infectious patient include health system-related factor such as delay in diagnosis. Factors that increase the progression of infection to disease are primarily endogenous (host related). Conditions which alter the immune response increase the risk of progression to disease with HIV coinfection, the most important of these. However at the population level impact of this risk factor could vary depending on the local prevalence of the HIV. Diabetes, alcohol, malnutrition, tobacco smoke, and indoor air pollution are factors which impact a larger section of the population and accelerate progression to TB disease. This paper aims to summarize the risk factors which contribute to TB infection and disease at both individual and population level.

## 2. Methods

The search strategy for this paper included searching PubMed, Medline, and EMBASE databases for known risk factors. Only English language papers were included in the search, and the searches were limited to studies of risk factors influencing TB infection and disease. Factors related to TB treatment outcomes such as mortality and default were not included. Broad search terms included the following: Tuberculosis, transmission, contacts as a MeSH or heading term as well as “tuberculosis,” “risk factors,” and “transmission,” as text words AND infectious diseases, Tuberculosis and risk factors as MeSH or subject terms and keywords. More focused searches were undertaken within specific Tuberculosis journals such as the International Journal of Tuberculosis and Lung Disease, the Indian Journal of Tuberculosis, the Bulletin of the World Health Organization, and the Indian Journal of Medical Research. Only major risk factors related to TB infection and disease were identified, relevant literature was reviewed, and factors influencing TB treatment outcomes were not included.

## 3. Summary of Specific Risk Factors

**Figure 1:**
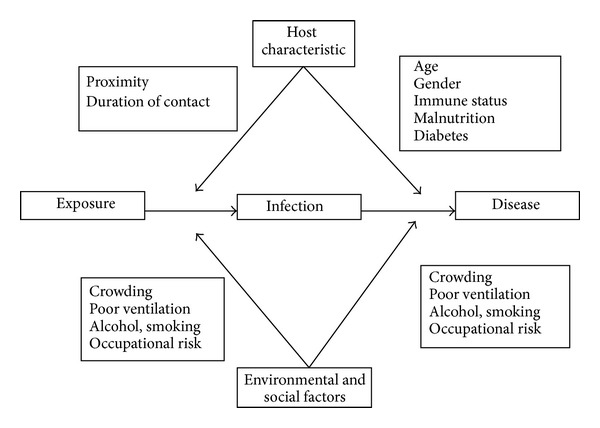
Risk factors for Tuberculosis infection and disease.

Figure 1 depicts the major characteristics which influence an individual's risk of contracting infection and disease, and the key risk factors are summarized below.

### 3.1. Factors Related to the Index Case

#### 3.1.1. Bacillary Load

Epidemiological studies conducted during mid-20th century have shown that smear positive cases are more infectious than the others [11, 12]. An untreated sputum positive patient can infect approximately 10 individuals per year, and each smear positive case can lead to two new cases of TB, at least one of which will be infectious [2, 13].

The concentration of bacilli in the sputum from a TB case is positively correlated with the infectivity of the TB patient. Espinal and colleagues, in their prospective study of 803 household contacts of 174 index TB patients in the Dominican Republic, administered 5 TU Tubersol PPD to contacts at baseline and followed them up at 2, 8, and 14 months to study the effect of HIV on the infectiousness of *Mycobacterium tuberculosis*. In their subanalysis they showed that the odds of TST positivity for contacts with an index case sputum smear grade 1–10 (bacilli per field) and >10 (bacilli per field) compared to 0 (bacilli per field) were 1.98 (CI = 0.75–5.23) and 5.88 (CI = 1.60–21.3), which clearly shows that being a contact of an index patient with higher-grade sputum was associated with a greater likelihood of having a positive TST [14].

Smear negative patients are expected to have reduced number of bacilli than smear positive patients but can also transmit infection [15] with experimental studies confirming that the infecting dose of *M. tuberculosis* bacilli can be as few as one to ten bacilli [16, 17]. Epidemiological studies conducted in USA, UK, and India (prevalence and incidence studies) comparing infection and disease rates clearly points that prevalence of infection and disease is higher among contacts of smear positive index cases than smear negative cases, but the rates were higher among smear negative compared to general population [18–27].

Behr et al. in their molecular study in San Francisco identified 71 clusters of patients infected with identical strains, and, out of 183 secondary cases in those clusters, 17% [28] were attributed to infection by smear negative patients [29] the remainder being smear positive. Similar studies conducted by Hernández-Garduño and colleagues in the Greater Vancouver regional district showed that the episodes of transmission from smear negative clustered patients ranged from 17.3 to 22.2% in the pulmonary and 25 to 41% among extra pulmonary group [15, 30]. Tostmann from the Netherlands [31] confirmed that 13% of the secondary-cases were attributable to transmission from smear negative patients. This indicates that patients diagnosed with a sputum-positive result are more likely to be infectious [10, 12, 28, 32], but smear negative cases also remain an important source of transmission.

#### 3.1.2. Proximity to an Infectious Case

Close contacts of infectious TB cases including household contacts and care givers/health care workers [33] are at a higher risk of becoming infected with *Mycobacterium tuberculosis *and development of primary active tuberculosis. Household contact studies among TB patients from early part of the 20th century [11, 34, 35] and large epidemiological surveys [20, 36–38] have established this effect. Morrison and colleagues performed a systematic review to determine the yield of household contact investigation [39]. Authors included 41 studies which were performed in 17 countries (49% in Africa, 29% in Asia, and 22% in Central and South America). The overall yield for all tuberculosis (bacteriologically confirmed and clinically diagnosed) was 4.5% (CI = 4.3–4.8) of contacts investigated; for cases with bacteriological confirmation the yield was 2.3% (CI = 2.1–2.5). Latent tuberculosis infection was found in 51.4% (CI = 50.6–52.2) of contacts investigated. However there was limitation, including the assumption that the transmission of infection and development of disease has occurred without biological evidence of organisms and the lack of community tuberculosis rates in the studies to see whether the findings are above the community average. TST was used in most studies for detecting LTBI, and the test is limited in its interpretation because of false positive and false negative results [40]. Subgroup analysis of sputum smear positive index cases showed that pooled yield for LTBI was 51.8% (CI = 50.9–52.8).

The risk of TB disease among individuals with LTBI (diagnosed as TST positive) relative to a person with no risk factors varies by several orders of magnitude. Several studies have asserted this finding. In two controlled clinical trials by Ferebee [41] examining the efficacy of treatment of LTBI among contacts of persons with active TB and among patients in mental hospitals, the tuberculin skin tests of 1472 participants in the placebo groups of the trials converted from negative to positive. Among persons whose tests converted, 19 developed disease in the first year of followup (12.9 cases per 1000 person-years) compared with 17 persons in the subsequent 7 years. of followup (1.6 cases per 1,000 person-years) [41]. A clear demonstration of the influence of proximity to an infectious case was shown in an airplane outbreak investigation. Passengers seated within two rows of the index TB patient were more likely to have positive tuberculin skin test compared to those in the rest of the section (30.8% versus 3.6%, RR = 8.5, CI = 1.7–41.3) [42].

Contact tracing efforts have therefore been targeted towards household members of TB cases based on the “stone in the pond” principle, with the probability of infection increasing with the proximity [43]. But the importance of community transmission of TB has been under debate for a long time; Blomquist [44] raised the issue of difficulties in defining contacts of a case and stressed the need for extending the definition of the term “contact” to a larger number of persons associated with each patient, implying that transmission occurs beyond the households. The number of cases of infection in a particular exposure group (defined by the closeness to the source case) is the product of the risk and the number of people in the group. Thus exemplifying the Rose axiom [45] “a large number of people at small risk may give rise to more cases than a small number of people at high risk”, there appears to be more cases of infection in the very large group of distant, low risk contacts than in the small group of close, high risk contacts. Conventional contact tracing generally identifies close, high risk contacts and therefore identifies only a minority of the infected contacts (20%), if higher than this, the circle of tracing needs to be widened [46].

The importance of casual contacts was noted in early epidemiological studies which showed that majority of older children with a positive TST reported no household contact with a source case and were therefore likely to have been infected in the community [19, 47–49]. Narain and colleagues in their retrospective analysis of a large household survey conducted in India [20] were able to show that, of the total persons infected in the community, only 2% belonged to case households, 7% belonged to suspect case households, and the remaining 91% of cases belonged to noncase households. The authors inferred that the zone of influence of an infectious case could extend to houses at least 10 lots distant [50]. Similar results were recorded by Radhakrishna et al. in their 15-year follow-up study of 253261 individuals in rural south India [26].

Molecular studies that identify the strain of the TB organisms have also confirmed the importance of casual transmission in both low- and high-incidence settings. In USA, Bishai and colleagues were able to show that there is an extensive transmission of TB occurring in the community. Of the 182 patients who had isolates available, 84 (46%) showed molecular clustering with 58 (32%) defined as being recently transmitted. Only 20 (24%) of 84 cases with clustered DNA fingerprints had epidemiologic evidence of recent contact. The remaining 64 (76%) cases without epidemiological links shared socioenvironmental risk factors for casual exposure to infectious TB cases (young age, homeless, alcohol, and drug use) and demographic features such as geographic aggregation in an area with inadequate housing [51]. These findings imply that TB continues to be propagated by casual recent transmission. Similar findings were found in other studies from low-incidence settings [52–56]. Similarly, Narayanan and colleagues have shown that 62% of patients (236/378) had identical strains in their large field survey in south India indicating a very high casual transmission [57]. Studies from other endemic settings like South Africa have confirmed this [58, 59].

These studies show that TB can be transmitted within a short period of contact [60], in nontraditional locations, and the opportunities for such interactions are abundant in an endemic setting with additional risk such as poverty, overcrowding, and high infection pressure [61]. Casual transmission is therefore a critical factor in TB dynamics in endemic settings [62].

### 3.2. Factors Related to the Individual

#### 3.2.1. Immunosuppressive Conditions

HIV coinfection is the most potent immunosuppressive risk factor for developing active TB disease [9]. Southern Africa has the highest prevalence of HIV infection and had the highest incidence of TB before the HIV/AIDS era. In the six southern African countries with adult HIV prevalence of more than 20%, the estimated TB case-notification rates are from 461 to 719 per 100 000 per year; by comparison, the notification rate in the USA was 5 per 100 000 per year [63]. HIV coinfection greatly increases the chances of reactivation of latent infection of TB [64] and increases the rapid TB progression following primary infection or reinfection with TB [5, 65–67]. Studies in countries with high HIV prevalence have also shown that spatial and temporal variation in TB incidence is strongly associated with the prevalence of HIV infection [9]. Individual studies conducted in both high- [68] and low-burden TB countries [69] have attributed increasing TB incidence to HIV infection.

HIV coinfection exacerbates the severity of TB disease while additionally TB coinfection accelerates HIV replication in affected organs including lungs and pleura [70]. Cell-mediated immunity is a crucial component in the host defence against *M. tuberculosis* that is weakened by HIV infection resulting in increased risks in reactivation of TB and commonly results in widespread dissemination causing EPTB. TB also accelerates HIV progression through increased systemic immune activation [71]. Therefore, coinfection leads to increases in the rate of disease progression and mortality [72, 73] among patients for multiple reasons.

Individuals with immune-mediated inflammatory disorders (IMID) are also known to be at increased risk of developing active TB, particularly after the use of tumour necrosis factor (TNF)—alpha inhibitors to treat a variety of autoimmune disease [74, 75]. Animal studies have shown that TNF is critical in host immune response in controlling a wide variety of bacterial, fungal, parasitic, and mycobacterial infection. Studies have shown that individuals are at increased risk for many of these infections, in particular for TB in areas with a high background prevalence of TB [75, 76]. Therefore screening for LTBI has been recommended before TNF-alpha inhibitor therapy is initiated. Both TST and IGRAs are being increasingly used to screen for LTBI, with IGRAs showing higher specificity. De Leon et al. evaluated TST and QFT responses in patients with RA and controls in Peru, a highly TB endemic region, where 80% of participants had a history of BCG vaccination. The proportion of patients testing positive for LTBI was significantly higher with QFT than with TST and more closely approximated that of the control group, suggesting that the IGRA was more sensitive than TST in detecting LTBI [77]. It is important to note that both the tests lack the ability to distinguish between latent TB infection and active disease; that is, none of the existing tests can accurately identify the subgroup that is at risk of progression to disease [78].

#### 3.2.2. Malnutrition

Studies have shown that malnutrition (both micro- and macro-deficiency) increases the risk of TB because of an impaired immune response [79–82]. TB disease can itself lead to malnourishment because of decreasing appetite and changes in metabolic processes [83]. The association between malnutrition and TB has been shown with BCG vaccine trials performed in USA during the late 1960s estimating that malnourished children are twice as likely to contract TB disease as their appropriately nourished peers [84]. The first National Health and Nutrition Examination (NHANES-1) and the NHANES-1 Epidemiological Follow-up Study (NHEFS) conducted during 1982–84 from the USA among adults reported an increased adjusted hazard of TB from six- to ten-fold [85] in malnourished individuals. However, Cegielski and McMurray reviewed the relationship between malnutrition and tuberculosis with the available ecological, epidemiological, and animal studies and commented that although evidence exists to relate malnutrition and TB, the risk relative to specific levels of malnutrition still needs to be defined [79].

#### 3.2.3. Young Age

Children are at higher risk of contracting TB infection and disease. Studies have shown that 60–80% exposed to a sputum smear-positive case became infected compared to only 30–40% who are exposed to a sputum smear-negative source case [48, 86–89]. Majority of the children less than 2 years of age get infected from the household source case, whereas, with children more than 2 years of age, majority of them became infected in the community. Household sputum positive source case is the single most important risk factor for children and remained an important contributor to infection up to 5–10 years of age [88]. Most of the disease manifestations develop within the first year following primary infection, identifying the first year following exposure as the time period of greatest risk. Children with primary infection before 2 years or after 10 years of age were at increased risk for disease development [90]. The highest risk for TB-related mortality following primary infection occurred during infancy. The risk declined to 1% between 1 and 4 years of age, before rising to more than 2% from 15 to 25 years of age [89, 90]. These findings provided the scientific basis for classical contact investigation practices, which focus on children less than 5 years of age in most developing countries and all household contacts in most industrialized countries.

#### 3.2.4. Diabetes

Diabetes has been shown to increase the risk of active TB disease [91, 92]. It is estimated that currently 70% of people with diabetes live in low- and middle-income countries [93], and the rates are steadily increasing in areas where TB is endemic, including India and sub-Saharan Africa [94]. A systematic review comparing 13 studies examining the association between diabetes and TB found that diabetic patients had about a threefold increased risk of developing TB when compared to those without diabetes [95]. Studies have also found poorer outcomes among diabetic patients with Alisjahbana et al. in their prospective study showing that patients with TB and DM had a 22.2% smear-positive culture rate at the end of treatment compared to only 6.9% of those without diabetes [96]. Another review on treatment outcomes among patients with DM and TB found that the risk of death was 1.89 times higher compared to those without diabetes, with the risk increasing to five times higher for those with DM after adjustment for potential confounders [97].

Biological evidence supports the theory that diabetes directly impairs the innate and adaptive immune responses, thereby accelerating the proliferation of TB. Animal studies showed a higher bacterial load among diabetic mice experimentally infected with *M. tuberculosis* [98]. Decreased production of IFN-*γ* and other cytokines diminished T-cell immunity [99] and reduced chemotaxis in neutrophils of diabetic patients [100] are thought to play a role in increasing the propensity of diabetic patients to developing active TB. A reverse association where TB can induce glucose intolerance and deteriorate glycaemic control in subjects with diabetes has also been identified [101]. Increasing rates of diabetes [102] in India could pose a great challenge for TB control in the future [103].

#### 3.2.5. Healthcare Workers

Healthcare workers (HCWs) are at increased risk of exposure to TB. A review by Seidler et al. showed that, among HCWs in high-income countries, the overall incidence of TB disease in the general population and native born HCWs was less than 10 and 25 per 100 000 per year [104]. Joshi and colleagues summarized evidence on the incidence and prevalence of latent TB infection (LTBI) and disease among HCWs in low- and middle-income countries. In their review of 51 studies the authors found that the prevalence of LTBI among HCWs was on 55% (CI = 33–79), the estimates of the annual risk of LTBI ranged from 0.5 to 14.3%, and the annual incidence of TB disease ranged from 69 to 5780 per 100 000 [33].

### 3.3. Socioeconomic and Behavioural Factors

Rapid urbanization [105, 106] witnessed in developing countries and socioeconomic status (SES) of individuals has also been shown to have influence on a person's susceptibility to infection. The TB burden follows a strong socioeconomic gradient between and within countries with the poorest having the highest risk [107, 108]. People with low SES are exposed to several risk factors discussed above (including malnutrition, indoor air pollution, alcohol, etc.) which increases their risk for TB. People with lower SES have a higher likelihood of being exposed to crowded, less ventilated places and have limited safe cooking practicing facilities. Marginalized populations including prisoners have a higher chance of getting infected with TB [109] mostly because of crowded living conditions and coinfection with HIV and injection drug abuse [110]. While smoking rates are higher among individuals belonging to lower SES, alcohol, HIV, and diabetes are not well correlated with lower SES [107].

#### 3.3.1. Tobacco Smoke

The association between smoking and TB has been studied in several systematic reviews [111–116]. Bates and colleagues, in their meta-analysis of 24 studies on the effects of smoking on TB, showed that the relative risk of TB disease (RR = 2.3–2.7) was high among smokers in comparison to nonsmokers and that there was clear evidence that smoking causes remained a risk factor for TB infection and disease, with additional risk of death in persons with active TB [114]. Lin et al. performed a systematic review and meta-analysis examining the role of smoking, indoor air pollution in TB from 38 studies. In their analysis of six studies specifically examining tuberculin reactivity among smokers, the pooled OR for latent TB infection (LTBI) was 2.08 (CI = 1.53–2.83) and 1.83 (1.49–2.23) at 5 and 10 mm TST cut-off points and the effect of smoking on LTBI remained even after adjustment for alcohol (OR = 1.76, CI = 1.43–2.16) [117]. The authors (with their pooled evidence showing increased risk for TB infection, disease, and deaths) commented that their data support a causal link between smoke exposure and an increased chance of acquiring TB, with the primary impact of smoking being to increase the risk of infection [117].

Biological explanations including impaired clearance of mucosal secretion [118], reduced phagocytic ability of alveolar macrophages [119, 120], and decrease in the immune response and/or CD4 + lymphopenia due to the nicotine in the cigarettes [120] have been given as reasons for increased susceptibility to pulmonary tuberculosis [112]. More recently Shang and coworkers in their animal study were able to demonstrate that exposure of mice to cigarette smoke followed by infection with *M. tuberculosis* results in a significant increase in the number of viable *M. tuberculosis* bacilli isolated from the lungs and spleen along with a decline in the adaptive immunity in the exposed mice [121].

#### 3.3.2. Alcohol

Alcohol has been recognized as a strong risk factor for TB disease [122], and a recent meta-analysis of molecular epidemiological studies has established alcohol as a risk factor for clustering (or recent transmission of TB) in both high- (OR = 2.6, CI = 2.13–3.3) and low-incidence countries (OR = 1.4, CI = 1.1–1.9) [123]. A systematic review of 3 cohort and 18 case control studies concluded that the risk of active tuberculosis is substantially elevated (RR = 2.94, 95% CI = 1.89–4.59) among people who drink more than 40 g alcohol per day and/or have an alcohol use disorder [122]. Reasons for increased risk include alteration in the immune system, specifically in altering the signalling molecules responsible for cytokine production [124].

#### 3.3.3. Indoor Air Pollution

In developing countries, the percentage usage of solid fuels for cooking is more than 80% [125]. Firewood or biomass smoke has been previously recognized as an independent risk factor for TB disease in case control studies conducted in India and Brazil [126–129]. Limited data on the mechanism by which biomass smoke causes chronic pulmonary diseases exists [130] however; animal studies have shown that acute wood smoke impaired macrophage phagocytic function, surface adherence [131], and bacterial clearance [132]. Also biomass combustion is shown to release large particulate matter (PM) such as carbon monoxide (CO), nitrogen oxide, formaldehyde, and polyaromatic hydrocarbons which can deposit deep into the alveoli and can cause considerable damage [133–135].

### 3.4. Demographic (Ethnic) Factors

#### 3.4.1. Indigenous/Aboriginal Population

Studies from Canada and Australia have shown that indigenous or aborigines are at a higher risk of TB than the nonaborigines [136–138]. Aborigines have a higher than average prevalence of predisposing risk factors for TB such as renal failure, diabetes, alcohol abuse, and smoking. In addition, socioeconomic factors such as overcrowding and poverty are known contributors to this burden [139]. A recent study showed that several aborigines in Canada had a gene deletion that may have predisposed them to developing active TB disease [140]. Clark and Vynnycky in their model predicted an increasing contribution of endogenous reactivation to total disease burden over time [138]. The high prevalence of latent infection, coupled with an increased risk of disease, may result in cases of reactivation disease in aboriginal communities.

### 3.5. Health System Issues

Evidences from China have demonstrated gains through strengthening health systems (by improving notification through web-based reporting), by which hospital referrals improved from 59% to 87% and the contribution of sputum positive pulmonary TB cases from hospitals doubled from 16% to 33% [141]. On the other hand, health system issues such as delays to diagnosis and treatment increase the duration in which active cases are infectious, thereby sustaining TB transmission [142]. Lin and colleagues in their cross-sectional study TB infection prevalence survey in southern China found that there was a positive association between the duration of delay to TB treatment and household infection rates [143]. The current passive case finding approach in the DOTS program is built upon the principle to treat infectious cases at the earliest to reduce the burden of infection or transmission in the community. This could be hampered by delay in diagnosis and treatment and may accelerate the transmission in the community [144, 145].

Table 1 provides summary estimates of relative risk for selected TB risk factors.

## 4. Conclusion

Screening for TB (to diagnose latent TB infection) and prophylactic therapy remain the most important tools to reduce the risk of progression to TB disease among high risk individuals (close contacts, HIV infected individuals, health care workers, etc.) and be considered in endemic countries to reduce the progression from infection to disease. Screening for latent TB also warrants highly sensitive and specific tools. The existing array (the newly available IGRAs) of diagnostic tests detect latent TB infection are highly specific but has reduced sensitivity [146]. Their inability to differentiate latent infection from disease and high operational costs makes them less than ideal tool for use in the developing world, where bulk of the TB infection and disease occurs.

HIV coinfection is the most important and potent risk factor for TB infection and disease. Interventions such as early HIV counselling and screening for TB patients and early diagnosis and initiation of antiretroviral therapy (ART) to coinfected individuals have all been shown to be effective in preventing TB disease [106].

In endemic countries, diagnosis and treatment (through DOTS) of smear-positive cases remains the key to TB control by reducing transmission from infectious cases. In addition to passive case-finding practices, early diagnosis of smear-positive cases can be improved through untargeted case-finding strategies in endemic countries [147]. Health system issues hampering this include a significant percentage (45% in countries like India) of TB patients accessing health care through the private sector [148]. Such patients are unaccounted for, and together with delay in diagnosis they may act as a constant reservoir for TB infection. Efforts to include private players (private practitioners, retail pharmacies, and laboratories) in TB control activities are therefore essential to curtail the epidemic.

The growing population (especially in countries like China and India) is likely to inflate the number of TB cases in future. Smoking rates are high among men in these endemic countries [143, 149], and, together with rising rates of diabetes [95], the risk of progression to TB disease will also increase. Interventions such as smoking cessation [150] and early screening for TB can be advocated, but the impact of these interventions in reducing TB risk remains negligible at population level [106].

Malnutrition and indoor air pollution are recognized risk factors which are confounded with the socioeconomic status of a setting. Rapid urbanization is shown to offset these components to an extent (by decreasing malnutrition rates and increased usage of clean fuels) [106], but increased awareness through IEC (information, education, and communication) activities should be considered. Efforts should also be made to collect risk factors data in routine surveillance for TB disease.

## Figures and Tables

**Table 1:** Relative risk, prevalence and population attributable risk of selected risk factors for TB.

Risk factor (reference)	Relative risk for active TB disease (range)^a^	Weighted prevalence, total population, 22 TB high burden countries^b^	Population attributable fraction (range)^c^
HIV infection	8.3 (6.1–10.8)	1.1%	7.3% (5.2–6.9)
Malnutrition	4.0 (2.0–6.0)	17.2%	34.1% (14.7–46.3)
Diabetes	3.0 (1.5–7.8)	3.4%	6.3% (1.6–18.6)
Alcohol use > 40 g/day	2.9 (1.9–4.6)	7.9%	13.1% (6.7–22.2)
Active smoking	2.6 (1.6–4.3)	18.2%	22.7% (9.9–37.4)
Indoor pollution	1.5 (1.2–3.2)	71.1%	26.2% (12.4–61.0)

^a^Range is equal to 95% confidence interval, except for malnutrition and diabetes.

^b^22 countries that together have 80% of the estimated global TB burden.

^c^Population attributable fraction = (prevalence × (relative risk − 1))/(prevalence × (relative risk + 1)).

Source: adapted from Lönnroth and Raviglione [151].
